# The Synergistic Effects of Structural Evolution and Attack Strategies on Network Matching Robustness

**DOI:** 10.3390/e27080847

**Published:** 2025-08-09

**Authors:** Xu Na, Junying Cui, Chang Su, Shimin Cai, Linyuan Lü

**Affiliations:** 1 Institute of Fundamental and Frontier Sciences, University of Electronic Science and Technology of China, Chengdu 611731, China; 2 Department of Physics, University of Fribourg, 1700 Fribourg, Switzerland; 3 School of Computer Science and Engineering (School of Cyber Security), University of Electronic Science and Technology of China, Chengdu 611731, China; 4 School of Cyber Science and Technology, University of Science and Technology of China, Hefei 230026, China

**Keywords:** matching energy, structural evolution, attack strategies, matching robustness

## Abstract

Research on network robustness has long focused on changes in the structure connectivity of networks under attacks, effectively depicting structural integrity while ignoring the exploration of functional integrity. When the core path of the network is attacked, even if it remains connected, the rapid increase in energy consumption may still trigger systematic risks. Existing studies mainly use random networks and scale-free networks as comparative models, which has become a classic research paradigm. However, real-world networks often exhibit mixed topological features. To address the above issues, this paper introduces the concept of energy from physics into bipartite networks and establishes an evaluation framework for assessing the synergistic effects of structural evolution and attack strategies on network matching robustness. We first introduce a structural parameter *u* to construct a structural evolution model, where the network’s minimal matching energy distribution evolves from topological heterogeneity to random features. When *u* approaches 0, edges with the minimal matching energy concentrate on a few candidates, manifesting scale-free network features. When *u* approaches 1, the uniform distribution of the minimum-matching-energy edges corresponds to random network features. We then design three types of edge attack strategies—minimum-energy (min-E), random-energy (ran-E), and maximum-energy (max-E) attacks—simulating the impacts of critical path destruction, uniform perturbation, and redundancy removal, respectively. In addition, we construct two evaluation indicators, the average matching energy and the matching retention rate. The results show that structural evolution significantly affects network matching robustness in a nonlinear manner. Different attack strategies also exert different influence on matching robustness. Furthermore, the findings reveal the synergistic effects of the two factors on network matching robustness. The synergistic effects of redundancy capacity and network structure on matching robustness are also explored. The research deepens the understanding of network matching robustness and provides a theoretical basis for resource allocation systems to combat network attacks.

## 1. Introduction

Research on network robustness [1,2,3,4] has long focused on changes in network structure connectivity (the largest connected component) under different attacks [4,5,6]. Drawing inspiration from percolation theory in physics [7,8,9], robustness is quantified through monitoring the dynamic decay of the giant component size with node or edge removal, thereby evaluating structural integrity. Targeted attacks (i.e., of nodes with highest degrees) and random attacks are two of the most studied attack strategies [10,11,12], widely revealing the vulnerability of scale-free networks to targeted attacks and their resilience to random attacks [13,14,15,16]. However, while existing studies effectively depict the structural integrity of networks, they ignore the exploration of functional integrity in resource allocation scenarios [8].

Research on network matching robustness is crucial to ensuring the integrity of network functions, especially in resource allocation scenarios, such as logistics networks [17,18,19,20] and transportation networks [21,22,23]. For example, on the Internet, accidental cuts in optical fibers lead to declines in communication efficiency. Similarly, in covert networks (including terrorist networks and criminal networks), the removal of important connections could cause a weakening of the resilience of criminal organizations, as these eliminated nodes are not easily replaceable [24,25]. The phenomenon of matching instability caused by such edge failures reveals the limitations of traditional connectivity indicators in evaluating functional integrity. When the optimal matching path fails, even if the network remains connected, the rapid increase in energy consumption caused by suboptimal paths may still trigger systematic risks.

Compared with traditional connectivity research, matching robustness extends the research perspective to the dynamic equilibrium mechanism of network micro-interactions, focusing on the sustainability of global optimal allocation under resource-constrained conditions [26,27,28]. Its core lies in exploring the dynamic impact of disturbances on the network’s energy landscape. Therefore, constructing a matching robustness evaluation framework holds important application value for designing disaster-resistant resource allocation networks.

Additionally, in the study of network robustness, employing random networks and scale-free networks as comparative models has become a classic research paradigm [29,30]. Despite sharing identical node counts and edge numbers, these two network types exhibit fundamental topological differences. Random networks follow a Poisson degree distribution with relatively uniform node connectivity, while scale-free networks display a power-law degree distribution characterized by a small number of highly connected hub nodes. However, this analysis framework exhibits two primary limitations. First, the framework overlooks the mixed topological features in real-world networks, which often integrate both power-law and random features [31,32,33,34]. Second, the fixed structural parameters in experimental designs fail to capture the dynamic evolution inherent in actual networks. These limitations hinder the exploration of the continuous evolution of network structures and limit the explanatory power of theoretical models for actual networks.

To address these issues, this study proposes a network matching robustness evaluation framework and explores the impact of edge attacks based on structural evolution on matching robustness. The main research contents include the following: (i) introducing a structural parameter *u* to construct a structural evolution model from the energy perspective of physics, where the matching energy distribution of the network evolves from topological heterogeneity (u→0) to random features (u→1); (ii) designing three types of edge attack strategies: minimum-energy (min-E), random-energy (ran-E), and maximum-energy (max-E) attacks; (iii) constructing two robustness evaluation indicators, the average matching energy and the matching retention rate. By monitoring the changes in the two indicators with the attack scale *f* and the structural parameter *u*, the impact of attack strategies and structural evolution on the matching robustness is revealed. Furthermore, the synergistic effects of redundancy capacity and network structure on matching robustness are also explored.

## 2. Model Description

### 2.1. Network Construction

We introduce the concept of energy from physics into bipartite networks composed of two distinct sets: *m*, individuals (e.g., users), and *n*, candidates (e.g., resources). Each individual i(i=1,2,…,m) ranks all candidates (j=1,2,…,n) and generates matching energies based on personalized preferences. By constructing the network structural parameter *u*(u∈(0,1]), we then establish an evaluation framework for assessing the synergistic effects of structural evolution and attack strategies on network matching robustness.

**Figure 1:**
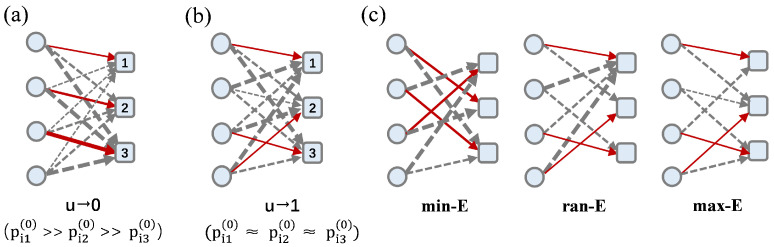
Schematic diagram of network construction. (**a**): The structural features of the network when u→0, and an optimal matching set. (**b**): The structural features of the network when u→1, and the optimal matching set. (**c**): The effects of the three attack strategies under u→1. The matching capacities of the three candidates are 1, 1, and 2, respectively. The size of the arrow represents the matching energy; the smaller the energy, the thinner the arrow. The gray dotted arrows represent all possible matches, and the red solid arrows represent the optimal matches.

The basic idea of the framework is as follows: Set the initial matching energy to each candidate as $e_{j}^{\prime }=j/n$, where j∈{1,2,…,n}. When *u* approaches 0, the network exhibits highly similar preferences converging to initial settings, reflecting high similarity in group preferences. At this stage, edges with minimal matching energy concentrate on a few candidates, manifesting ‌scale-free network features [35,36] (Figure 1a). With increasing values of *u*, the distribution of the minimal matching energy gradually changes to randomization, showing mixed features. ‌When *u* approaches 1‌, the network shows typical random features with completely random individual preferences and uniformly distributed minimum-matching-energy edges, corresponding to random network features (Figure 1b). Based on the framework, we first establish a matching energy distribution function and then construct a matching energy matrix reflecting collective preferential behaviors. Specifically, based on the Boltzmann distribution in physics, the matching energy distribution is defined as follows:

(1) $$p_{ij}^{\left(0\right)}\left(u\right)=\frac{exp(log\left(u\right)*e_{j}^{\prime }/\lambda )}{\sum_{j=1}^{n}exp\left(log\left(u\right)*e_{j}^{\prime }/\lambda \right)},$$

where $p_{ij}^{\left(0\right)}\left(u\right)$ represents the initial matching probability between individual *i* and candidate *j* under the parameter *u*, while $e_{j}^{\prime }$ represents the initial matching energy of candidate *j*. The parameter λ(>0) modulates the exponential decay rate of preference concentration. The larger the λ value, the more dispersed the distribution. As *u* approaches 0, log(u)→−∞; the exponential decay is sharpened and the optimal matching energy of the group is forced to concentrate. Meanwhile, as *u* approaches 1, log(u)→0; the decay rate slows, flattening the distribution to uniformity.

Information entropy is a concept that quantifies the uncertainty of a system [37,38], originating from the concept of entropy in physics. A higher value of information entropy indicates greater uncertainty in the system. Based on the matching energy distribution, we can calculate the information entropy for different network structures as follows:

(2) $$H\left(u\right)=\frac{1}{m}\sum_{i=1}^{m}H_{i}\left(u\right)=-\frac{1}{m}\sum_{i=1}^{m}\sum_{j=1}^{n}p_{ij}^{\left(0\right)}\left(u\right)log\left(p_{ij}^{\left(0\right)}\left(u\right)\right),$$

In order to compare the information entropy under different network structures, we normalize it as follows:

(3) $$H_{norm}\left(u\right)=\frac{H\left(u\right)-H_{min}\left(u\right)}{H_{max}\left(u\right)-H_{min}\left(u\right)},$$

where $H_{max}$ represents the maximum information entropy value, which corresponds to the random network characteristics with parameter *u* = 1. All candidates have the same probability of being selected, that is,

$$H_{\max}\left(u\right)=H\left(1\right)=\log\left(n\right).$$

$H_{min}$ represents the minimum information entropy value, which corresponds to the scale-free network characteristic with parameter *u* = 0.01; $H_{min}\left(u\right)=H\left(0.01\right)$. At this time, there is a significant difference in the probability of options being selected.

We then generate preference lists based on the distribution using the Plackett–Luce model [39,40], which is a probabilistic ranking method that balances deterministic preferences and randomness. The procedure iteratively samples the candidates without replacement. To be specific, for each individual *i*, it initializes its preference sequence $E_{i}$ as an empty set, and at each time step, t(t=1,…,n), candidate $j^{t}$ is sampled without replacement from the remaining set with a probability equal to $p_{ij}^{\left(t-1\right)}\left(u\right)$. The matching energy between the individual *i* and the selected candidate $j^{\left(t\right)}$ is defined as follows:

(4) $$e_{ij}^{\left(t\right)}=\frac{t}{n},$$

where *t* represents the preference ranking value assigned by individual *i* to candidate $j^{\left(t\right)}$, while *n* represents the total number of candidate options. The energy value corresponds to the position in the preference ranking. The higher the ranking, the lower the energy value and the higher the matching stability. For example, the candidate selected by *i* at the first time step is its favorite, corresponding to the highest stability, and thus has the lowest matching energy 1/n.

Then, the probability distribution for the remaining candidates is renormalized as follows:

(5) $$p_{ij}^{\left(t\right)}\left(u\right)=\frac{p_{ij}^{\left(t-1\right)}\left(u\right)}{1-\sum_{s=1}^{t}p_{ij}^{\left(s-1\right)}\left(u\right)},$$

where $\sum_{s=1}^{t}p_{ij}^{\left(s-1\right)}\left(u\right)$ represents the cumulative probability of all the selected candidates. It is guaranteed that during each step, the sum of probabilities across all available candidate options equals exactly 1. This mathematical framework maintains probabilistic consistency throughout the selection process.

By recording the candidates selected by the individual *i* at each time, *t*, along with their corresponding matching energies, $e_{ij}=e_{ij}^{\left(t\right)}$, we obtain the preference sequence $E_{i}$ for the individual, which is formally defined as

(6) $$E_{i}=\left[\begin{array}{cccc} e_{i1} & e_{i2} & \ldots & e_{in} \end{array}\right].$$

Repeating this process for all individuals, we obtain the collective energy matrix *E*, which is defined as follows:

(7) $$E=\left[\begin{array}{c} E_{1}\\ E_{2}\\ \vdots \\ E_{m} \end{array}\right]=\left[\begin{array}{cccc} e_{11} & e_{12} & \ldots & e_{1n}\\ e_{21} & e_{22} & \ldots & e_{2n}\\ \vdots & \vdots & \ddots & \vdots \\ e_{m1} & e_{m2} & \ldots & e_{mn} \end{array}\right].$$

The matrix corresponds to a fully connected bipartite network, where each individual *i* ranks all candidates according to personalized preferences. We then model the problem as a flow network and apply the minimum-cost maximum-flow algorithm from Python’s ortools library to yield the optimal matching set M={(i,j)} for the network [41,42]. The energy values $e_{ij}$ in the matrix serve as unit flow costs, and the matching capacity of each candidate is set to $c_{j}$.

In this study, we mainly focus on resource-scarce matching scenarios with limited candidate capacity. We first explore the impact of structural evolution and attack strategies on matching robustness in the simplest case, with all capacities being one. Then, we investigate the synergistic effect of a slight increase in capacity and structural evolution on matching robustness, where the capacity of each candidate equals 3, 5, 7, and 9, respectively.

### 2.2. Attack Strategies

To systematically evaluate the matching robustness of networks under different adversarial conditions, we designed three edge-based attack strategies: min-E attacks, ran-E attacks, and max-E attacks (Figure 1c). The min-E attacks are designed to evaluate network vulnerability to targeted removal of critical low-energy edges, reflecting attacks on high-preference connections. This strategy prioritizes disrupting the most stable matching paths. The ran-E attacks aim to assess the system’s resilience against uniformly distributed disruptions, mimicking random failures or non-targeted adversarial actions. The removal probability is uniform across all edges, independent of energy values. The max-E attacks aim to evaluate the role of redundant high-energy edges in maintaining network robustness, simulating attacks on less-preferred but important backup paths.

The attack scale is defined as *f* (f∈[0,1)), and the implementation step is that for each individual *i*, the attack removes the f∗n edges with the smallest energy, randomly selected energies, and the largest energy, respectively. After each attack, the updated energy matrix $E_{a}$ is fed into the minimum-cost flow solver to recompute the optimal matching set *M*.

The fully connected bipartite graph is selected as the initial state primarily to establish an idealized limit reference frame. This approach eliminates interference from the initial topological heterogeneity, ensuring that experimental differences stem entirely from the distinct attack strategies. By progressively increasing the attack ratio *f*, the impact of the three edge-based attack strategies on the optimal matching energy of the network can be accurately quantified.

### 2.3. Evaluation Indicators

Two indicators are defined to reveal the matching robustness of networks under edge attacks in this subsection. The first indicator is the average matching energy, defined as

(8) $$\bar{e}=\frac{1}{\left|M\right|}\sum_{(i,j)\in M}e_{ij},$$

where $e_{ij}$ represents the matching energy of individual *i* and candidate *j* in the set of optimal solutions *M*, and |M| represents the number of matching pairs in *M*. A lower $\bar{e}$ indicates a higher matching stability. This metric captures the dynamic impact of attacks on matching quality by quantifying the incremental increase in network energy resulting from matching conflicts. When optimal matches are disrupted, suboptimal matching produces elevated network energy levels. This energy variation directly captures the extent of matching quality degradation.

The second indicator is the matching retention rate, defined as

(9) $$p=\frac{\left|M\right|}{|M_{max}|},$$

where $|M_{max}|$ is the maximum number of matching pairs when the attack scale is zero (f=0). A higher p represents a higher matching stability. This metric quantifies the proportion of matching preserved at a macro level after attacks.

## 3. Results

**Figure 2:**
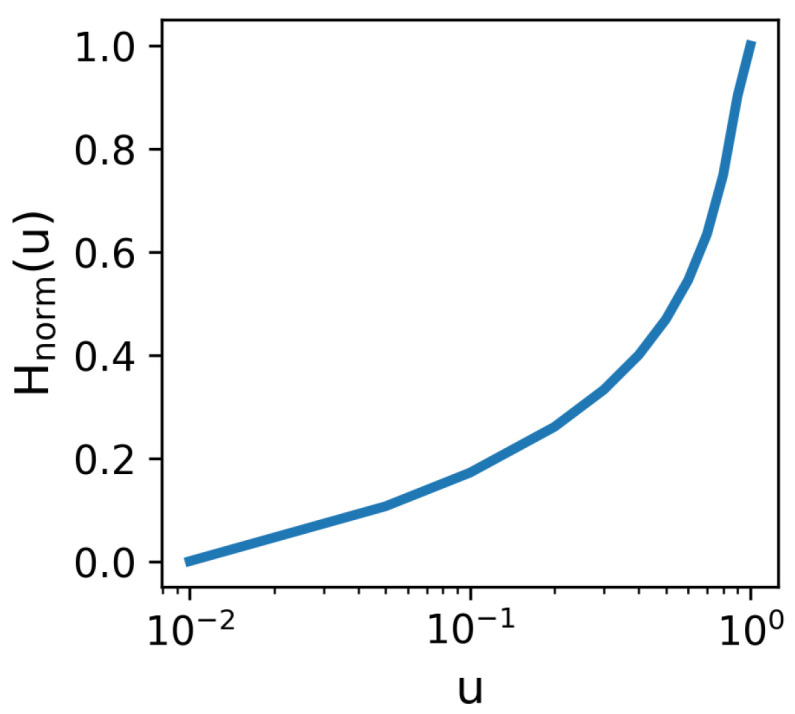
The relationship between network structure parameter *u* and information entropy $H_{norm}\left(u\right)$. The structural parameter’s range is [0.01, 1].

The results indicate that the structural parameter of the network *u* is proportional to the entropy value $H_{norm}\left(u\right)$, and the larger the *u*, the faster the $H_{norm}\left(u\right)$ increases (Figure 2). This shows that when the optimal matching energy distribution of the network shows complete topological heterogeneity, the individual matching preferences become highly predictable, resulting in a lower uncertainty in the optimal matching of the network—that is, lower information entropy. Conversely, when the energy distribution is completely random, all candidate options are chosen with equal probability, leading to a higher uncertainty—that is, to a higher information entropy.

**Figure 3:**
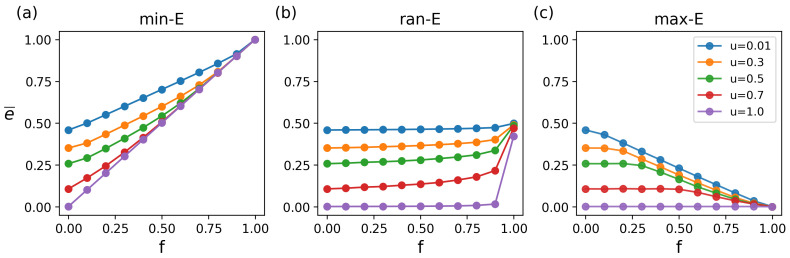
The impact of attack strategies with ratio *f* on average matching energy $\bar{e}$. (**a**) The min-E attacks. (**b**) The ran-E attacks. (**c**) The max-E attacks. The structural parameters are 0.01, 0.3, 0.5, 0.7, and 1.0 respectively. The figures from left to right (**a**–**c**) represent min-E, ran-E, and max-E, respectively. In all cases, the parameters of the network are $m=10^{3}$, $n=10^{3}$, and λ=0.03, and simulations were conducted for 50 iterations.

The results show significant differences in the impact of different attack strategies on average matching energy (Figure 3). Specifically, the min-E attacks severely destabilize networks, with the energy ($\bar{e}$) increasing sharply as attack intensity (*f*) increases (Figure 3a). Even low-intensity attacks trigger significant fluctuations. This is mainly because the attacks preferentially remove low-energy (i.e., critical) connections, destroying the optimal matching path and triggering a cascading effect. In contrast, under the ran-E attacks, the network exhibits strong robustness. Even for large attack scales (f<0.8), the energy remains relatively stable (Figure 3b). This is because the random edge removal uniformly distributes damage without causing cascading effects. Under the max-E attacks, the network shows moderate robustness, as removing high-energy redundant edges minimally affects core connections (Figure 3c).

**Figure 4:**
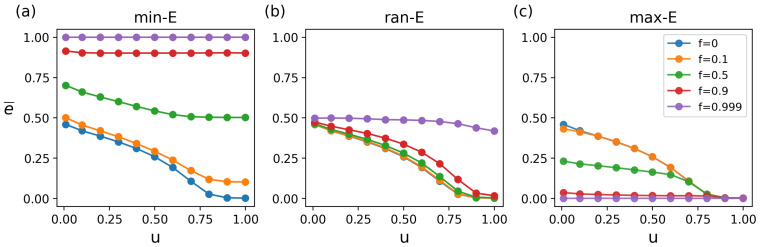
The impact of structural parameter *u* on average matching energy $\bar{e}$. (**a**) The min-E attacks. (**b**) The ran-E attacks. (**c**) The max-E attacks. The attack ratios are 0.0, 0.1, 0.5, 0.9, and 0.999 respectively.

In addition, the results reveal that the network structure significantly affects the matching robustness through nonlinear regulation (Figure 4). Specifically, under the unattacked condition, the average matching energy values corresponding to the structural parameters *u* = 0.01, 0.2, 0.4, 0.6, 0.8, and 1.0 are 0.459, 0.387, 0.310, 0.192, 0.0266, and 0.002, respectively. The lower the energy, the higher the matching robustness. When the network is dominated by topological heterogeneity (u→0), which indicates a sensitive structure state, increasing *u* significantly enhances network robustness. In scenarios such as logistics optimization and resource allocation, the optimal matching of nodes highly depends on the availability of low-energy edges. The high spatial aggregation of a small number of key connections intensifies the resource competition among the nodes. This mechanism is similar to the cascading failures observed in traditional node attacks targeting central nodes [8,43,44,45]. When u→1, the network enters a stability state, where a further increasing *u* will not improve the robustness. In this state, low-energy edges are widely distributed in the topological space. When under attack, the dispersion of key paths effectively avoids local overload, thereby significantly improving the matching robustness, aligning with the percolation resilience observed in random networks [29,30].

**Figure 5:**
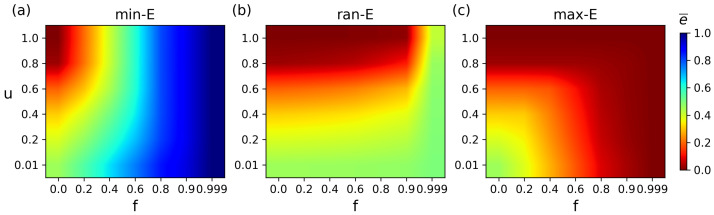
Synergistic effects of attack strategies with ratio *f* and structural parameter *u* on average matching energy $\bar{e}$. (**a**) The min-E attacks. (**b**) The ran-E attacks. (**c**) The max-E attacks.

Figure 5 shows the synergistic impact of *f* and *u* on the average matching energy under different attack strategies. Redder colors indicate lower energy (higher stability), while bluer colors denote higher energy (lower stability). Under min-E attacks, the network exhibits obvious dual-stage impact features: when under low-intensity attack (f<0.3), the change in matching energy arises from the synergistic interaction between the attack scale and the topological structure. However, as the attack scale increases to f=0.6, the change in energy is mainly driven by the attack scale, and the influence of the structure can be neglected (Figure 5a). At this high attack level, in varied topological structures with *u* values of 0.01, 0.2, 0.4, 0.6, 0.8, and 1.0, the corresponding energy values are always greater than 0.6. This shows a significant difference compared to the unattacked condition, reflecting the vulnerability of matching robustness to targeted attacks. In contrast, under ran-E attacks, the network displays a structure-dominated property where attack scale minimally affects matching energy, as structural differences primarily dictate energy variations. In contrast, when the attack ratio f=0.6, the energy values in different structures are 0.465, 0.402, 0.334, 0.227, 0.051, and 0.004, respectively (Figure 5b). The low degree of energy change compared to the unattacked condition indicates that the difference in network topology becomes the decisive factor. Only in the case of extreme attacks (f>0.9) does the structural influence become ineffective. Under max-E attacks, the network also shows dual-stage impact features, and its change trend is similar to that when under min-E attacks (Figure 5c).

**Figure A1:**
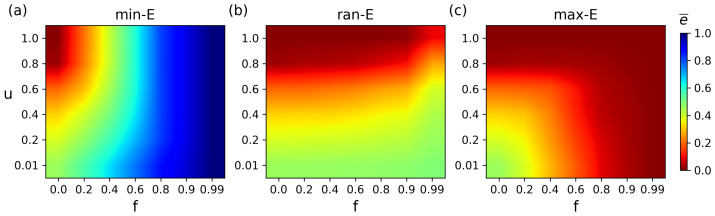
Synergistic effects of attack strategies with ratio *f* and structural parameter *u* on average matching energy $\bar{e}$ under the condition where n<<m. (**a**) The min-E attacks. (**b**) The ran-E attacks. (**c**) The max-E attacks. The parameters of the network are $m=10^{3}$, $n=10^{2}$, and λ=0.03, and simulations were done for 50 iterations.

It should be emphasized that this study mainly focuses on matching problems in resource-scarce scenarios; that is, the number of candidates does not exceed the number of individuals. The research findings are based on conditions where the user sets and the resource sets are of equal size (n=m). To validate the generalizability of the results, we further investigated conditions where the number of candidates is significantly smaller than the number of individuals (n≪m). Experimental results demonstrate that the changing trend of network average matching energy remains highly consistent across varying resource/user ratios (Figure A1). This consistency validates the generality of our research findings.

Notably, relevant studies show that under the same complex network size, the network structure (random network vs. scale-free network) has almost no significant impact on connectivity robustness under the random attack strategy. However, our study reveals that under random attacks, the structure still has a significant effect on network matching robustness. This is due to the connectivity focusing on the existence of edges between nodes, whereas matching stability concerns the global optimality of interconnection costs. Structural differences, therefore, lead to divergent stability, especially under resource scarcity. This highlights the need for functional integrity metrics beyond traditional connectivity measures.

**Figure 6:**
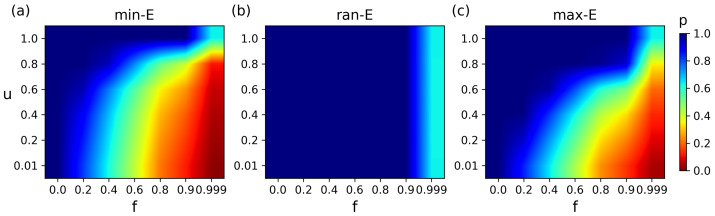
Synergistic effects of attack strategies with ratio *f* and structural parameter *u* on matching retention rate *p*. (**a**) The min-E attacks. (**b**) The ran-E attacks. (**c**) The max-E attacks.

**Figure A2:**
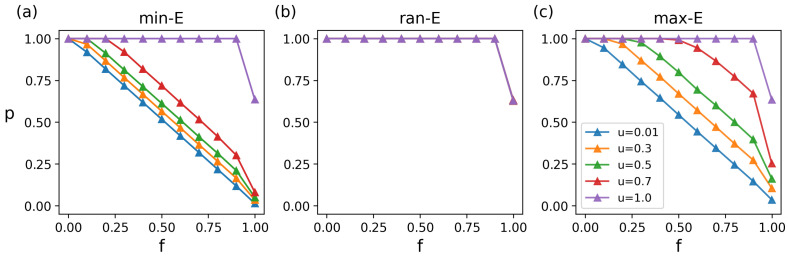
The impact of attack strategies with ratio *f* on matching retention rate *p*. (**a**) The min-E attacks. (**b**) The ran-E attacks. (**c**) The max-E attacks. The structural parameters are 0.01, 0.3, 0.5, 0.7, and 1.0 respectively.

**Figure A3:**
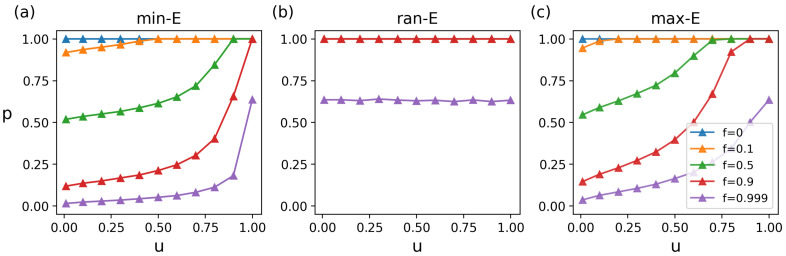
The impact of structural parameter *u* on matching retention rate *p*. (**a**) The min-E attacks. (**b**) The ran-E attacks. (**c**) The max-E attacks. The attack ratios are 0.0, 0.1, 0.5, 0.9, and 0.999 respectively.

We also explore the impact of *f* and *u* on the matching retention rate (*p*) under different attack strategies, and its changing characteristics are similar to the changes in $\bar{e}$. Specifically, under min-E attacks, *p* declines dramatically as *f* rises (Figure 6 and Figure A2), and structural changes further affect *p* (Figure A3). Figure 6a intuitively shows the changing trend of *p*. The larger the *f* and the smaller the *u*, the greater the decrease in *p*. Under ran-E attacks, the *p* remains stable even at high *f* and different *u* values, showing strong robustness (Figure 6b). Under max-E attacks, the changing trend is similar to with min-E attacks (Figure 6c). The main difference is that as *u* increases, *p* is more stable against *f*.

**Figure 7:**
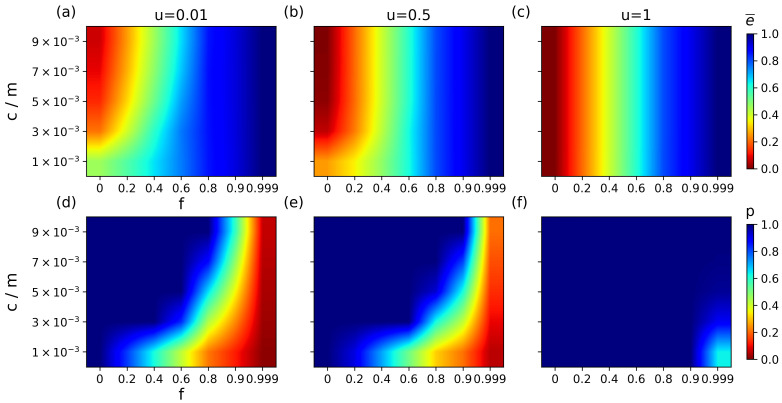
Synergistic effects of structural parameter *u* and candidate capacity *c* on matching robustness under min-E attacks. The structural parameters are 0.01, 0.5, and 1 respectively, and the matching capacity varies from 1 to 3, 5, 7, and 9 m ($=10^{3}$), representing the number of individuals in the network. The figures from (**a**–**c**) and (**d**–**f**) represent the changes in average matching energy and matching retention rate, respectively.

**Figure A4:**
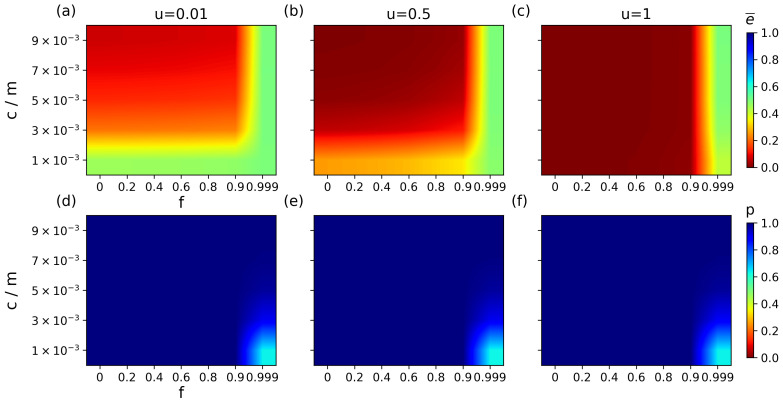
Synergistic effects of structural parameter *u* and candidate capacity *c* on matching robustness under ran-E attacks. The structural parameters are 0.01, 0.5, and 1 respectively, and the matching capacity varies from 1 to 3, 5, 7, and 9. The figures from (**a**–**c**) and (**d**–**f**) represent the changes in average matching energy and matching retention rate, respectively.

**Figure A5:**
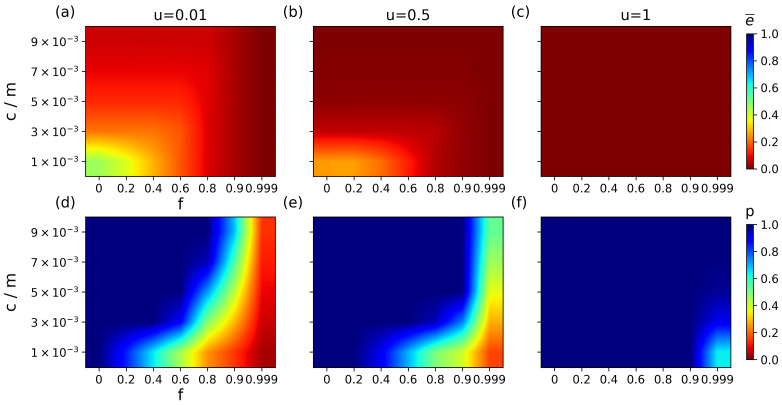
Synergistic effects of structural parameter *u* and candidate capacity *c* on matching robustness under max-E attacks. The structural parameters are 0.01, 0.5, and 1 respectively, and the matching capacity varies from 1 to 3, 5, 7, and 9. The figures from (**a**–**c**) and (**d**–**f**) represent the changes in average matching energy and matching retention rate, respectively.

Furthermore, we investigate the synergistic effect of a slight increase in matching capacity *c* and structural evolution on matching robustness. The impact of capacity on matching robustness depends on the structural features of the network. Specifically, when the network is dominated by topological heterogeneity (small *u*), a slight increase in capacity can significantly improve robustness under min-E attacks, with a lower energy and a higher matching rate (Figure 7). This change shows the same trend with different attack strategies (Figure A4 and Figure A5). This verifies the positive feedback mechanism between redundant capacity and robustness [46,47], where redundant capacity effectively disperses the local load impact caused by attacks by providing alternative matching paths. It shows that the degree of influence of redundancy on robustness decreases as the structural parameter increases. When the network is dominated by random features, changes in capacity will no longer work. This is because both the uniformity of the network structure and the redundant capacity play a role in dispersing the key paths and reducing the damage of targeted attacks to the network as a whole. Therefore, when structural uniformity is large enough, additional capacity redundancy will not come further into play.

## 4. Conclusions

This study introduces the concept of energy from physics into bipartite networks and constructs a structural parameter for networks in order to investigate network robustness from a matching perspective. By formalizing the structural evolution model and the three attack strategies, this work establishes an evaluation framework for assessing the synergistic effects of structural evolution and attack strategies on network matching robustness. Attack strategies exhibit significant differences in their impact on network matching robustness. The structural evolution also influences network matching robustness in a nonlinear manner. Furthermore, the findings reveal the synergistic effects of the two factors on network matching robustness. Specifically, the network exhibits obvious dual-stage impact features under min-E attacks and max-E attacks, whereas the network displays a structure-dominated property under ran-E attacks. The findings also emphasize the synergistic role of topological heterogeneity and redundancy management in enhancing matching robustness. The research deepens the understanding of network matching robustness and provides a theoretical basis for resource allocation systems to combat network attacks.

In addition, this study offers insights for the design and optimization of real-world network systems. In network cybersecurity protection, it highlights the necessity of establishing a multidimensional robustness evaluation framework. This framework should simultaneously monitor changes in network connectivity and network matching energy under attacks. When confronting attacks, the system should maintain topological connectivity while employing optimization algorithms to select repair paths with minimal matching energy increments. Such a dynamic mechanism will effectively reduce network operational costs. In network architecture design, implementing resilience recovery strategies against critical edge attacks is essential. For critical edges, systems should deploy redundant connections characterized by low energy consumption. When they are compromised, the system can automatically switch to preset alternative low-energy pathways, thereby significantly enhancing the network’s robustness against attacks.

## Data Availability

No new data were created or analyzed in this study. Data sharing is not applicable to this article.

## Abbreviations

The following abbreviations are used in this manuscript:

min-E	Minimum-energy
ran-E	Random-energy
max-E	Maximum-energy
